# Risk factors for the development of stifle injuries in canine agility athletes

**DOI:** 10.3389/fvets.2024.1335939

**Published:** 2024-02-14

**Authors:** Nina R. Kieves, Abigail Shoben, Arielle Pechette Markley

**Affiliations:** ^1^Department of Veterinary Clinical Sciences, College of Veterinary Medicine, The Ohio State University, Columbus, OH, United States; ^2^Division of Biostatistics, College of Public Health, The Ohio State University, Columbus, OH, United States

**Keywords:** agility, canine, stifle injury, risk factor, canine athlete

## Abstract

**Objectives:**

Our aim was to determine risk factors for developing stifle injuries in canine agility athletes. We hypothesized that increased weight, increased frequency of competing, and greater number of runs/day would increase risk.

**Study design:**

Internet based survey, *n* = 4,197.

**Methods:**

Individuals with at least one dog who had competed in agility in the past 3 years were eligible. Injury history was defined as an injury to the stifle that kept the dog from participating in agility for >1 week. Logistic regression models were used to estimate associations between variables of interest and injury history.

**Results:**

Handlers of 216 dogs reported a history of injury. The majority were cranial cruciate ligament (CCL) injury (101/216), and patella luxation (40/216). In the final model, five variables were independently associated with odds of stifle injury (in addition to age). Heavier dogs (adjusted for height), Border Collies, male dogs neutered <10 months, female dogs spayed before their first heat cycle, handlers aged 18–24, and teeter contact behavior were associated with increased risk.

**Conclusion:**

Heavier dogs were more likely to report injury, but there was no association with injury and increased number of competition days, or runs/day. The Border Collie breed was at the highest risk of injury. There was substantial risk for stifle injury with early spay/neuter. Additionally, a significant increase in risk of injury was reported for younger (18–24) vs. older handlers (65+). Athlete fitness level, conformation, and genetic predisposition to injury may play the most significant role in the development of injury.

## 1 Introduction

Canine agility is a highly physical sport with frequent abrupt turns taken at high speed, coupled with running and jumping, and the need to navigate obstacles that change in elevation. Courses include numerous obstacles that dogs must complete, with the goal of completing the course in the shortest time with no errors. The physical demands of these activities place significant stress on the dog’s musculoskeletal system, particularly on the joints. Injury rates of up to 42% have been reported in the literature for dogs competing in agility (1–5). Thoracic limb injury is most commonly reported including injury to the shoulder and paws (1–3). However, one study reported pelvic limb injuries to be more common than thoracic limb injuries (4).

One of the most common debilitating orthopedic injuries an athlete can suffer is a knee injury. In humans, injury is often suffered during athletic events that require similar lateral cutting motions such as dogs undergo while competing in agility. Stifle injury in dogs competing in agility has been reported as high as 13% of injuries in one study (1), and 10% in another, with over 75% of these being classified as severe (6).

Stifle disease is a significant health concern for agility dogs, particularly if the injury is to the cranial cruciate ligament (CCL), as it is documented that these dogs have a low chance of returning to sport (7). Treatment of CCL disease is also costly financially to the handler/owner and carries with it a significant loss of competition time. Given the impact that injury to the stifle can have on a dog’s agility career, our aim was to evaluate risk factors associated with developing stifle injury while participating in canine agility competitions. We hypothesized that increased weight, along with increased frequency of competing and increased number of runs per day of competition would increase risk of stifle injury.

## 2 Materials and methods

### 2.1 Study design

An internet-based survey, in English, was distributed via the internet with approval by the institutional review board at The Ohio State University using Qualtrics survey software (Qualtrics; Provo, UT). Specifics of this survey and results were previously published (1, 8). Briefly, individuals with at least one dog who had competed in agility in the past 3 years were eligible to complete the survey.

Stifle injury history was defined as an injury to the stifle that kept the dog from participating in agility for over a week. All participating owners were asked several questions about demographic variables (for handler and dog), training variables (e.g., age starting training each obstacle, method used for training contact and weave obstacles, trained contact obstacle behavior), and competition history (frequency of competing, runs per competition day, frequency of national and international competition, and frequency of competing on various surfaces). Owners reporting a stifle injury were asked additional follow up questions regarding the injury.

### 2.2 Statistical analysis

Statistical analysis was performed with commercially available software (Stata version 15.1, StataCorp, College Station, TX). Logistic regression models were used to estimate possible associations between variables of interest and stifle injury history. All models were adjusted for dog age to account for differences in exposure time for injury history. For the three pre-specified variables of interest (height and weight together, number of competition weekends per year, and typical number of runs per day), we tested each for an association in models that only adjusted for dog age. Given the lack of information on risk factors for stifle injury in the literature, we also considered a broader set of candidate variables via a stepwise model building process. All candidate variables were first assessed for univariate association with injury; variables significant at *p* < 0.20 were kept for future model building. Then three models were built via backward selection using blocks of variables (demographic, training, and competition factors). Variables that were significant in the stepwise model building at *p* < 0.20 were kept for consideration in the final model. The final model was built via backward selection until all included variables were associated with injury at *p* < 0.05.

## 3 Results

Complete demographic data related to this study population has been previously published (1). The most common breeds represented were Border Collie (*n* = 1,052), mixed breed (*n* = 616), and Australian Shepherd (*n* = 312). Mean age of the dogs at the time of survey was 6.3 ± 2.6 years.

Of the 4,197 dogs in the sample, 216 (5.2%) had a history of stifle injury. Nearly half (46.8%) of dogs reporting a stifle injury reported a cranial cruciate ligament (CCL) rupture (101/216). Other stifle injuries reported by more than 5 dogs were: medial luxating patella (26, 12.0%), arthritis (16, 7.4%), lateral luxating patella (14, 6.5%), medial collateral ligament sprain (13, 6.0%), lateral collateral ligament sprain (12, 5.6%), and caudal cruciate rupture (6, 2.8%). The exact diagnosis was reported to be unknown for 37 dogs (17%). Owners reported that they knew or suspected the injury occurred in competition or practice for 30% of dogs (65/214), while 45% (*n* = 97) said it did not and 24% (*n* = 52) were unsure. These percentages were nearly identical for the 101 CCL rupture injuries reported: 32% (32/101) in competition or practice; 46% (46/101) not in competition or practice, and 23% (23/101) were unsure.

In models adjusting only for dog age, body characteristics (height and weight) were associated with stifle injury risk, with heavier dogs (of the same height) having a higher odds of stifle injury (OR: 1.27 per 10 pounds (4.5 kilograms) heavier; 95% CI: 1.12 to 1.44) and taller dogs (of the same weight) having lower odds of stifle injury (OR: 0.73 per 4 inches (10.2 centimeters) shorter; 95% CI: 0.59 to 0.91). Number of trial weekends per year was not associated with odds of stifle injury (*p* = 0.99) with all groups (<5 weeks per year up to 26+ weekends per year) having similar odds of injury. Similarly, number of runs per trial day was not associated with odds of stifle injury (*p* = 0.46).

**Figure 1:**
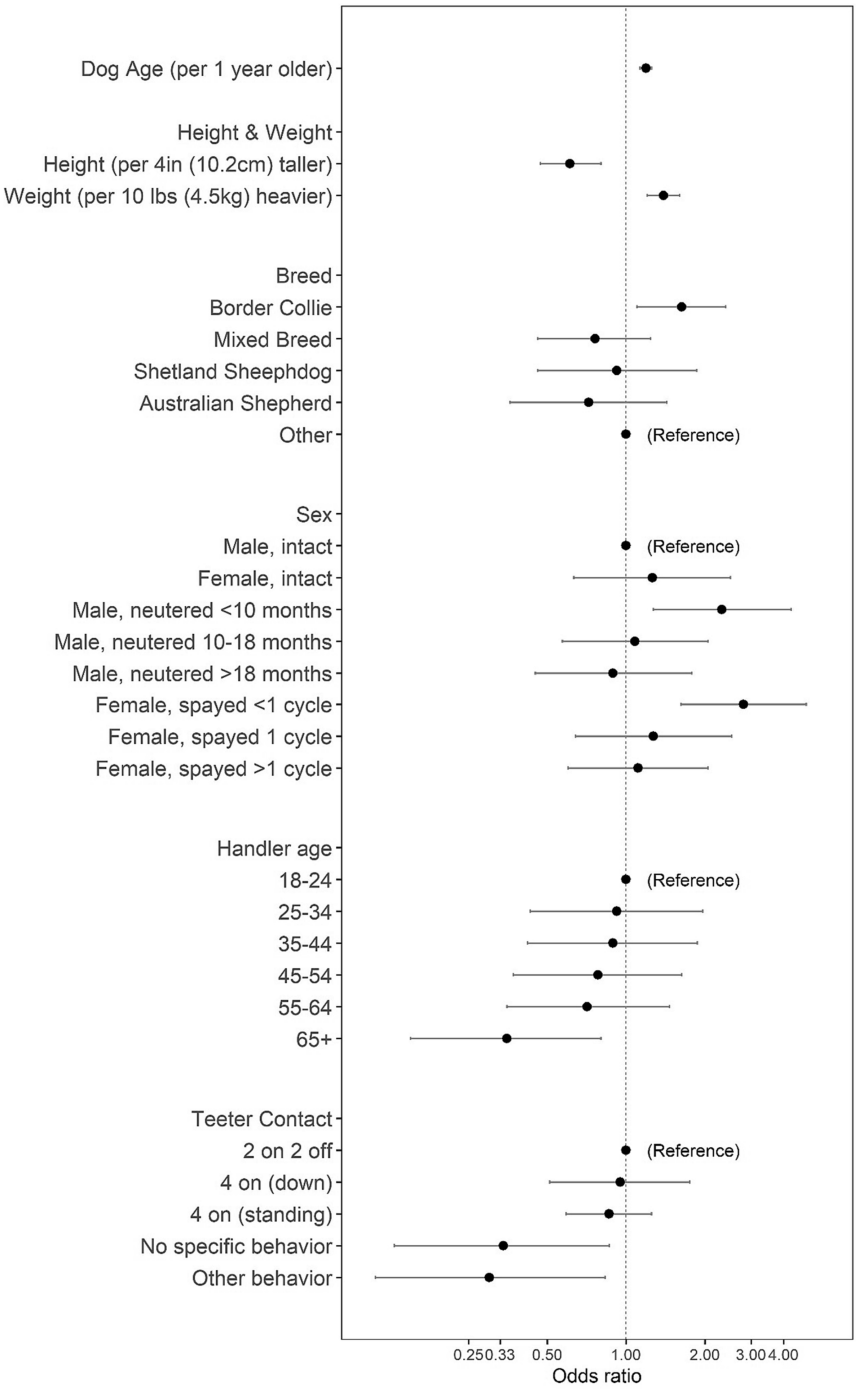
Graphical display of the odds ratios for history of stifle injury from the final model built using stepwise selection.

In the final model built via stepwise selection (Table 1; Figure 1), five variables were independently associated with odds of stifle injury (in addition to age). Body characteristics (height and weight) were associated with stifle injury, with taller dogs having lower odds of developing a stifle injury and heavier dogs (adjusted for height) having increased odds of injury. The other four variables in the final model were breed, spay/neuter status, handler age, and teeter contact behavior. Among breeds, Border Collies were at higher risk and there were minimal differences noted among other breed groups. Male dogs neutered before 10 months and female dogs spayed before their first heat cycle had markedly higher reported rates of stifle injury compared to all other sex/neuter groups that had generally similar odds of stifle injury. There was a notable decrease in odds as handler age increased, with the highest odds of injury observed among dogs of the youngest handlers (18–24) and the lowest among dogs of handlers aged 65 years and older (OR: 0.35). For the teeter contact, dogs that were either not trained to perform a specific behavior at the end of the teeter or dogs performing a different behavior than the most common training options, had lower risk of reporting a history of stifle injury.

**Table 1:** Odds ratios from final model built using stepwise selection.

	Adjusted OR final model	*p*-value
Dog Age (per 1 year older)	1.19 (1.13, 1.25)	<0.0001
Height & Weight		<0.0001
Height (per 4 in (10.2 cm) taller)	0.61 (0.47, 0.80)	
Weight (per 10 lbs. (4.5 kg) heavier)	1.39 (1.20, 1.60)	
Breed		0.029
Border Collie	1.63 (1.10, 2.40)	
Mixed Breed	0.76 (0.46, 1.24)	
Shetland Sheepdog	0.92 (0.46, 1.86)	
Australian Shephard	0.72 (0.36, 1.43)	
Other	REFERENCE	
Sex		0.0001
Male, Intact	REFERENCE	
Female, Intact	1.26 (0.63, 2.50)	
Male, Neutered <10 months	2.32 (1.27, 4.26)	
Male, Neutered 10–18 months	1.08 (0.57, 2.05)	
Male, Neutered >24 months	0.89 (0.45, 1.78)	
Female, Spayed <1 cycle	2.81 (1.62, 4.88)	
Female, Spayed 1 cycle	1.27 (0.64, 2.53)	
Female, Spayed >1 cycle	1.11 (0.60, 2.05)	
Handler current age		0.041
18–24	REFERENCE	
25–34	0.92 (0.43, 1.96)	
35–44	0.89 (0.42, 1.87)	
45–54	0.78 (0.37, 1.63)	
55–64	0.71 (0.35, 1.46)	
65+	0.35 (0.15, 0.80)	
Teeter contact		0.039
2 on 2 off	REFERENCE	
4 on (down)	0.95 (0.51, 1.75)	
4 on (standing)	0.86 (0.59, 1.25)	
No specific behavior	0.34 (0.13, 0.86)	
Other	0.30 (0.11, 0.83)	

## 4 Discussion

While we found the expected association between heavier dogs and stifle injury history, there was no association with report of injury and an increased number of competition days, or runs per day. Therefore, our hypothesis was partially accepted. The lack of association of increased injury risk with increased number of competition days and/or runs may indicate that there truly is no association with increased agility-specific activity and stifle injury. It could also be a reflection of overall training load. If a dog trains substantially but does not compete often, their overall time under load could be similar to a dog that trains little but competes frequently. Prospective training data would need to be collected over the duration of a dog’s competitive lifespan to elucidate such information. Our group has previously evaluated the effect of training load on injury risk and found that dogs who train very little, or for >120 h per week had a higher risk of developing injury than dogs who trained an intermediate amount (9). However, this study evaluated overall risk of injury while competing in agility, not specific types of injury. The lack of association observed between increased injury risk and number of competition days may also reflect a scenario in which dogs who are not as fit, or have had a stifle injury, are not entered into as many competitions or runs during competition by their handler as compared to those dogs who are healthy. Without training data, and prospective evaluation of fitness levels, we cannot assess these factors.

In the adjusted model, the Border Collie breed was at the highest risk of reporting stifle injury as compared to other breeds, with no other common agility breed (Australian Shepherd, Shetland Sheepdog, or mixed breed) showing an increased risk of injury. Border Collies competing in agility have been previously reported as having a higher risk factor of injury overall (1). In a previous report evaluating CCL injury risk in the general population, Border Collies were the 29th most common breed to report CCL injury (10). There is likely geographic variability in injury given that Engdahl found Boerboel’s and Dogo Canario to have the highest rate of development of CCL injury in Sweden (11). We were unable to specifically evaluate risk for breeds known to be considered high risk for CCL injury (10) as they were not highly represented in this survey. Therefore, it is possible, due to the sample size for some of these breeds (i.e., Labrador Retriever), that we were unable to capture a significant increase in risk in these populations. Furthermore, we evaluated overall stifle risk injury, not only CCL injury.

Further study is needed into why Border Collies competing in agility may be at a higher risk of developing stifle injury including CCL injury than the general population of Border Collies. It may be related to genetics of the breed, breed conformation, or their speed and high drive during competitions, which have become even more complex and challenging over time. Contrary to our finding here that the Border Collie is at higher risk for developing stifle disease, a recent publication evaluating risk factors associated with CCL injury in agility dogs did not find the Border Collie to be at a higher risk for injury than other breeds (12). Our finding of increased risk for Border Collies developing stifle injury was especially high after adjusting for dog height and weight when compared to other breeds evaluated. This adjustment was not done in the Sellon et al. study (12), which could also explain the differences seen between these two studies. Again, our study assessed risk for all types of stifle injury, of which CCL injury was the highest reported injury, but this difference could also account for the increased risk found here.

The majority of stifle injuries reported here were CCL injuries, which pose a substantial potential loss of career for these athletes, as only 65% of agility dogs return to sport following tibial plateau leveling osteotomy (TPLO) surgery for treatment of CCL injury (7). In human medicine a higher percentage of patients are reported to return to sport at 80% following anterior cruciate ligament (ACL) reconstruction surgery. However, only 65% of them return to their pre-injury level of performance and only 55% return to a competitive level of sport (13). This is despite studies showing that dogs undergoing TPLO have a return to near normal ground reaction forces as soon as 150 days after surgery (14, 15). Therefore, it is unknown why dogs may not return to competition despite returning to “normal” function as evaluated by force plate. Given these data, clients should be warned that when CCL injury is sustained, return to sport, particularly return to highly competitive levels of sport, may not be possible. Setting appropriate expectations early in the process of recovery should improve clinician-client relationships. It is currently unknown why such a low percentage of dogs return to sport. This could indicate that competitive agility dogs do not have the standard expected outcome with the stabilization technique elected, or it may be the handler’s choice to no longer compete with that dog following injury and treatment, as opposed to the dog’s lack of physical ability to perform agility activities. Furthermore, we do not know what degree of osteoarthritis these dogs had, nor what their meniscal status was, both of which likely play a role in ability to return to full competitive level of agility. We also cannot determine based on the survey data collected what the nature of the CCL injury in terms of a possible traumatic tear vs. the more commonly sustained degenerative injury to which dogs are prone. Histopathology of the CCL would be required to determine the ultimate cause of injury, and this is not commonly performed.

It may be that conformation and genetic predisposition to CCL injury play the most significant role in the development of this injury (16). In human ACL injury, the most common mechanism of injury is sudden pivoting or cutting maneuver which often occur during sports such as soccer, basketball, and football. Non-contact injury is also reported, with risk factors associated with tearing of the ACL including sex (female > male), and numerous bone morphologic characteristics such as lateral femoral condylar ratio, notch width index, and lateral posterior tibial slope (17). In dogs, one study found an association between tibial anatomical-mechanical axis angle and CCL injury (18). Due to the nature of this survey, conformational and genetic factors were not able to be assessed. The survey did ask whether the injury was thought to have occurred during competition or training, with approximately 30% of owners reporting that the injury (either CCL or other) occurred during agility training or competition. However, the complex etiology of CCL disease specifically makes it challenging to determine whether stifle injuries reported during training and competition were truly acute, traumatic injuries, or progression of previous chronic partial tears that were a result of other underlying risk factors. A study evaluating the cause of CCL injury in field trial dogs found owners to be inaccurate in their understanding of and assessment of how the injury occurs (i.e., traumatic vs. degenerative) (19). Therefore, we cannot say if any of the injuries reported were truly traumatic while actively participating during agility, or in fact whether they might have occurred regardless of the dog’s participation in agility activities. For instance, dogs that sustained a CCL injury may have done so even without participation in agility at all during their lifetimes.

An increased risk of stifle injury with increased body weight was identified. Sellon and Marcellin-Little, also reported an association between increasing dog weight and CCL injury in a population of agility dogs (12). Obesity has been found to be associated with CCL injury (10, 20–22), and obesity is more common in neutered dogs (6). Heavier weight, as adjusted for height, was found to be a risk factor for injury in this survey, which may support those previous findings (13). Athlete fitness level may also play a substantial role in the development of CCL injury. Sellon and Marcellin-Little reported that agility dogs performing routine core strength and balance exercises had lower risk of reported CCL injury (12). Muscle activation has been proposed as a contributing factor to the development of CCL injury in dogs (23), as well as humans (24).

Early spay/neuter was associated with a substantial increase in risk for stifle injury. Previous reports have suggested that early spay/neuter may increase risk of CCL injury (10, 20, 21), and the majority of stifle injuries reported here were CCL injury, which may have helped drive this result. Previous reports have also shown an overall increased risk in the development of orthopedic disease in larger dogs with early spay/neuter (25). Given the small number of other injuries reported, we were unable to statistically evaluate CCL injury as compared to other stifle injuries to better assess which risk factors are most associated with which specific stifle injuries. This should be prospectively studied in the future to determine if the risk factors reported here influence specific stifle injuries (CCL vs. patella vs. other). Ultimately, additional prospective work is needed to best assess what factors may decrease risk of developing CCL injury in agility dogs including whether targeted strengthening programs may help prevent CCL and stifle injury in general in dogs.

Similar to previous studies (26, 27), we found a significant increase in risk of injury history in dogs with younger handlers (18–24) as compared to older handlers (particularly 65+). Younger handlers are likely to be less experienced and may not be as precise with their handling, which could result in more reactionary movements from the dog, such as more sudden changes in speed and turning. It is also possible that they may have started agility as a hobby with their pet dog, who may not be the fittest or conformationally sound and thus be more prone to injury. Additionally younger handlers may not pick up on subtle signs of injury as well as older, more experienced handlers, therefore allowing their dog to continue competing and ultimately leading to progression of injury. The finding that dogs either not trained to perform a specific behavior at the end of the teeter, or dogs performing a different behavior than the most common options, have a lower risk for reporting a history of stifle injury may be related to handler experience, particular training techniques, or specific biomechanical forces incurred during teeter performance.

Inherit limitations of a survey include potential inaccuracies due to participant recall and handler-reported data without confirmation by a veterinarian. Self-selection bias may also result in the sample selection not being representative of the total agility dog population. Furthermore, we were unable to assess risk factors for specific stifle injuries due to small numbers reported here. Lastly, while injuries are reported in dogs performing agility, we could not elucidate whether the injuries occurred specifically due to agility training or competition or occurred secondary to performing agility. The injuries might have occurred in these dogs regardless of whether they actively participated in agility or not.

This survey provides insight into potential risk factors associated with all stifle injuries in agility athletes. The potential risk for stifle injury with early spay/neuter should be further explored. While we have begun to have a better understanding of musculoskeletal injuries due to increased availability of advanced imaging, there remains a lack of understanding of the kinetics and kinematics of dogs participating in sport, and how it relates to injury risk. Such studies are needed to enable us to make appropriate recommendations for prevention of injury. This particularly true regarding CCL injury given its significant impact on athletic capabilities following injury.

## Data availability statement

The raw data supporting the conclusions of this article will be made available by the authors, without undue reservation.

## Author contributions

NK: Conceptualization, Investigation, Methodology, Project administration, Supervision, Writing – original draft, Writing – review & editing. AS: Data curation, Formal analysis, Methodology, Writing – review & editing. AM: Data curation, Formal analysis, Methodology, Writing – review & editing, Investigation, Project administration.
